# Epidemiological and economic burden of *Clostridium difficile* in the United States: estimates from a modeling approach

**DOI:** 10.1186/s12879-016-1610-3

**Published:** 2016-06-18

**Authors:** Kamal Desai, Swati B. Gupta, Erik R. Dubberke, Vimalanand S. Prabhu, Chantelle Browne, T. Christopher Mast

**Affiliations:** Evidera Inc., London, UK; Merck & Co., Inc., Kenilworth, NJ USA; Washington University School of Medicine, St. Louis, MO USA; Merck & Co., Inc, 770 Sumneytown Pike, 19486 West Point, PA USA

**Keywords:** Cost, Community, Hospital, Long-term care, NAP1

## Abstract

**Background:**

Despite a large increase in *Clostridium difficile* infection (CDI) severity, morbidity and mortality in the US since the early 2000s, CDI burden estimates have had limited generalizability and comparability due to widely varying clinical settings, populations, or study designs.

**Methods:**

A decision-analytic model incorporating key input parameters important in CDI epidemiology was developed to estimate the annual number of initial and recurrent CDI cases, attributable and all-cause deaths, economic burden in the general population, and specific number of high-risk patients in different healthcare settings and the community in the US. Economic burden was calculated adopting a societal perspective using a bottom-up approach that identified healthcare resources consumed in the management of CDI.

**Results:**

Annually, a total of 606,058 (439,237 initial and 166,821 recurrent) episodes of CDI were predicted in 2014: 34.3 % arose from community exposure. Over 44,500 CDI-attributable deaths in 2014 were estimated to occur. High-risk susceptible individuals representing 5 % of the total hospital population accounted for 23 % of hospitalized CDI patients. The economic cost of CDI was $5.4 billion ($4.7 billion (86.7 %) in healthcare settings; $725 million (13.3 %) in the community), mostly due to hospitalization.

**Conclusions:**

A modeling framework provides more comprehensive and detailed national-level estimates of CDI cases, recurrences, deaths and cost in different patient groups than currently available from separate individual studies. As new treatments for CDI are developed, this model can provide reliable estimates to better focus healthcare resources to those specific age-groups, risk-groups, and care settings in the US where they are most needed. (Trial Identifier ClinicaTrials.gov: NCT01241552)

**Electronic supplementary material:**

The online version of this article (doi:10.1186/s12879-016-1610-3) contains supplementary material, which is available to authorized users.

## Background

*Clostridium difficile* (*C. difficile*) is a gram positive, spore-forming bacterium with sequelae ranging from mild diarrhea to life-threatening pseudomembranous colitis and even death [1, 2]. Persons at increased risk for *C. difficile* infection (CDI) have advanced age, recent antibiotic exposure, proton pump inhibitor use, long length of stay in healthcare settings, serious underlying illness, or immunocompromised conditions [3, 4]. Around 20 % to 30 % of patients will go on to have a recurrent episode of CDI [5]. In addition to the aforementioned risk factors, prior recurrences and healthcare acquired infection further increase the probability for subsequent recurrences [6, 7]. CDI most commonly recurs within a week after treatment cessation but can recur up to 6 to 8 weeks later.

A changing epidemiology of CDI in the United States (US) and other industrialized countries has been observed since the early 2000s resulting in a substantial increase in the incidence and severity of CDI, especially in patients over 65 years of age [6–9]. Hospitalizations with CDI as a principal diagnosis more than doubled in the US from 33/100,000 population to 115/100,000 between 1993 and 2008 [10]. However, since 2009 a levelling-off or decline of CDI incidence has been observed in the hospital setting [11], which may be attributable to increased awareness, leading to improved environmental controls, hygiene or implementation of more comprehensive prevention strategies. A recent study reported an incidence of 48 and 93 per 100,000 population for community-associated and health care-associated infections, respectively in the US in 2011 [12]. These high incidence rates of disease may be due in part to the emergence of a highly virulent strain of *C. difficile*, known as restriction enzyme analysis type BI, North American Pulsed Field type 1 (NAP1), or polymerase chain reaction ribotype 027. The BI/NAP1/027 strain has spread widely after first outbreaks in the eastern US, Canada, and the United Kingdom [13]. While CDI is well-recognized as an infection that is hospital-acquired, recent studies have increasingly highlighted the importance of CDI in other settings such as the community or long-term, outpatient care settings [1, 12, 14].

Estimates of epidemiological and economic burden of CDI have relied on independent studies that vary considerably in their designs, populations, and methodologies or have reported this burden secondary to other objectives, making comparison and interpretation difficult [10, 12, 15–24]. One published source of CDI incidence in the US reports 336,600 hospitalizations in 2009 but does not include community-acquired cases or provide estimates in higher risk groups [10]. Recurrence rates have been variously reported between 3 % and 65 % (with most estimates falling between 20 % and 30 %), depending on period of observation, and risk factors such as episode, age, strain, or other factors [3, 4, 10, 19, 21, 25, 26]. Mortality estimates are similarly highly heterogeneous with estimates ranging from 3 % to 36 % [4, 6, 8, 10, 19, 22, 26–33]. The uncertainty in such estimates makes it difficult to understand the true CDI burden in the U.S. There is limited information on CDI burden occurring in the community, although more data are emerging in this area, as is the evidence of burden in specific high-risk patients, like the immunocompromised or organ transplant recipients. Economic burden estimates available from multiple sources vary widely, capturing different healthcare cost components in the management of CDI or cost perspective [34–36]. There are limited estimates of economic burden that include indirect costs such as productivity losses.

Our objective was to obtain detailed estimates of the epidemiological and comprehensive economic burden of CDI in the US in 2014 using consistent methodology across different risk groups, healthcare settings and community.

## Methods

### Model

We developed a decision-analytic model incorporating CDI epidemiology, natural history and costs for patients of different ages, and comorbid conditions from several healthcare settings and the community for multiple years, drawing primarily on published literature to inform model parameters. Focusing on year 2014, the model estimates the annual number of initial and recurrent CDI cases in the US by following the population of susceptible patients in the US and simulating the natural history of CDI from the index infection and up to six recurrences in the subsequent 12 months. The model also estimates severe episodes, colectomies, treatment outcomes, deaths, and annual direct and indirect costs for the population. The model was developed in Microsoft Excel (Version 14.0, 2010, Microsoft Corporation, Redmond, WA).

### Settings and patient population

We simulated the incidence of CDI in four settings: hospitals, long-term care facilities (LTC), long-term acute care (LTAC) hospitals, and the community. Since this study included modeled incidence of CDI, ethics review was not required. Patients in each setting were stratified into eight age groups: <1 year, 1–4 years, 5–9 years, 10–17 years, 18–44 years, 45–64 years, 65–84 years, and 85+ years. The hospital population was further divided into 5 groups at high risk for CDI: patients with hematopoietic stem cell transplantation (HSCT), inflammatory bowel disease (IBD), solid organ transplant (SOT), severe chronic kidney disease (CKD) and immune-compromised patients, plus a sixth reference group admitted to hospital not belonging to the previous risk groups. More detail of settings and risk groups is given in Additional file 1: Appendix A.

### Natural history of CDI

**Fig. 1:**
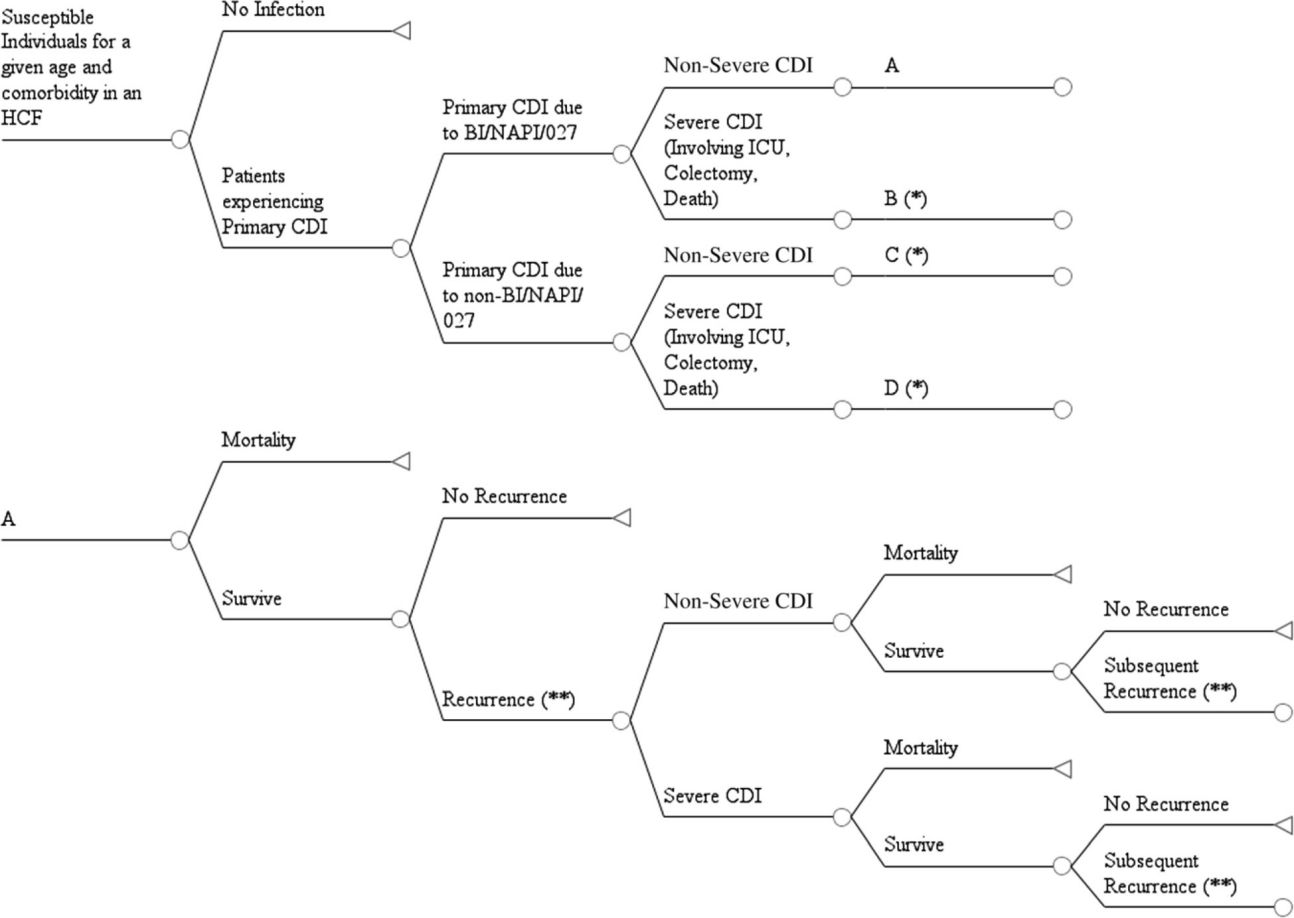
Diagram of decision-analytic model of CDI showing natural history for a given age and risk group in a healthcare setting. (*) Pathways for B, C, D are the same as for A, but downstream parameters for mortality and recurrence depend on strain. (**) Pathway for second and subsequent recurrences follows the same natural history as first recurrence although the probability of 2^nd^ + recurrence is greater than 1^st^

The layout of the decision-analytic model is given in Fig. 1 and follows the susceptible population for one year. The model consists of chance nodes with associated probabilities that determine the proportion of the population that is likely to follow a given natural history pathway. From the initial chance node representing the susceptible population, a proportion will experience incident CDI. Among these incident CDI cases, a certain fraction is likely caused by NAP1/B1/027 versus other strains. This strain dichotomization was chosen given the epidemiologic importance of NAP1/BI/027 in terms of mortality and recurrence. A proportion of persons experience a severe episode (intensive care unit care, colectomy or death attributable to CDI infection). Patients with the NAP1 strain have a higher chance of an incident severe episode and death vs. patients with non-NAP1 strains. As CDI can be a recurrent disease, a proportion of patients cleared of the index infection experience a future episode. We assumed that recurrent episodes were caused by the same strain as the initial case. Survivors of the first recurrence may experience up to five subsequent recurrences, where the probability of the second recurrence and beyond is greater than the first. Recurrence probabilities additionally depend on strain and age [4, 10, 25].

### Epidemiologic outcomes

The model generates epidemiologic outcomes for the number of initial CDI cases, recurrent cases according to order of the episode (first, second, third, fourth or higher); number of severe CDI cases and deaths attributable to CDI and all-cause deaths. Outcomes are presented specific to age, risk group and healthcare setting for year 2014.

### Economic burden outcomes

Economic outcomes for primary and recurrent CDI included direct costs for hospitalization, pharmacotherapy, healthcare professionals, and medical procedures, as well as indirect costs due to productivity losses, number of hospitalization days, and lost productivity. Costs were estimated using a societal perspective; direct and indirect costs for the hospital and community were included. Direct costs were computed for primary and recurrent cases. The quantities of each healthcare resource utilized in the management of a case of CDI were identified from the literature, and multiplied by unit costs of each healthcare resource. The cost of a CDI episode depended on its severity, whether it was primary or recurrent, whether one or more courses of antibiotic treatments were required and the patient’s age. Indirect costs included productivity losses associated with inability to work and were estimated as a product of national average age-specific wage rate, labor force participation, and duration of the episode. All cost projections from the model were presented in 2014 US dollars.

### Model parameters and data sources

Additional file 1: Appendix B gives key demographic, epidemiologic and economic model parameters, their base-case values and sources. Demographic parameters specified the annual number of susceptible pools of individuals in the hospital, LTC, LTAC, and community settings. Epidemiological parameters included initial CDI incidence, probability of incident cases due to NAP1/BI/027 strain, case severity, mortality, and recurrence. Economic parameters specified costs and quantities of direct and indirect costs associated with CDI.

Crude CDI incidence in the hospital setting combining all age and risk groups was derived from an analysis of primary and secondary diagnoses of CDI (ICD-9 008.45) from the Healthcare Cost and Utilization Project (HCUP) 2009 Nationwide Inpatient Sample [10]. In order to avoid over-estimating episodes, we used HCUP State Inpatient Databases (SID) [37] to estimate incidence of CDI as a primary or secondary diagnosis using unique patient identifiers for the year 2010 amongst all susceptible individuals; this analysis generated a correction factor that was applied to the 2009 data so that incidence rates were derived from individual patients that were only counted once. Crude incidence in hospital was also further adjusted downward to exclude 16.2 % and 25.5 % of CDI cases that occur in LTC and community, respectively, who are transferred to hospital to further correct for any over counting of cases [22, 38].

In order to assign a relative risk of CDI incidence occurring in hospital to specific age- and risk-groups, we separately utilized HCUP SID analyses using the same International Statistical Classification of Diseases (ICD) -9 codes which defined the susceptible individuals in the hospital risk groups. In these separate analyses, patients who belonged to HSCT, IBD, SOT, CKD, and immune compromised were at 7.8, 4.1, 3.6, 4.5, and 4.9 times, respectively, greater risk of CDI (all ages combined) relative to crude CDI incidence (data not shown). We excluded CDI infection in patients <1 year old since cases in this age group typically represent colonization rather than true disease. Similarly, we assumed HSCT is unlikely to occur in individuals >85 years old; hence susceptible patients in this age and risk group were set to 0.

Incidence of CDI in LTC, LTAC and community settings and other epidemiological parameters such as *C. difficile* strain, severity, mortality, and recurrence were based on the literature and assumptions described in Additional file 1: Appendix B. Additional file 1: Appendix B also lists the healthcare resources consumed in the management of CDI cases, productivity losses, and their unit costs sources. In the base-case, non-severe cases of CDI incurred on average 3 bed-days of hospitalization in the general ward (GW) [30, 35, 39]. It was assumed that a severe case of CDI required 12 bed-days in the general ward plus 11 bed-days in an ICU [39]. CDI cases managed in the community assumed one general practitioner contact per episode. Costs were also estimated for other procedures including colectomy, abdominal computerized axial tomography (CT) scans, peripheral intravenous line insertion, endoscopy, and toxin tests. Further details are included in Additional file 1: Appendix B. Productivity losses arising from CDI were calculated according to the product of duration of the episode; the mean earnings by age of the patient, and labor participation by age, obtained from the Bureau of Labor Statistics (BLS) [40]. Cost inputs from years earlier than 2014 were inflated using the CPI from BLS.

All CDI-related deaths were costed as severe CDI cases, while non-CDI related deaths assumed costs of non-severe CDI.

### Base-case and sensitivity analysis

We presented the base-case results for estimated initial and recurrent cases of CDI by setting, age, and risk factor in 2014. One-way sensitivity analyses were performed by varying those parameters which were most influential on the annual number of primary and recurrent CDI cases and total economic cost of CDI. Further details are provided in Additional file 1: Appendix C.

## Results

### Disease burden

In 2014, 606,058 episodes of CDI were expected nationwide in 439,237 patients who would subsequently have 166,821 recurrent episodes (see Table 1). A significant number of CDI patients, 150,648 or 34.3 %, were estimated to have CDI from community exposure. There were 112,152 (25.5 %) cases in LTC residents, and 170,229 (38.7 %) had overnight stays in an acute care hospital. Less than 2 % of cases occurred in LTAC settings.

**Table 1:** Model-based estimates of CDI burden in the United States for 2014 by setting and risk group. Figures in parentheses are proportions of row totals

	Hospital – Ref Group	Hospital - HSCT	Hospital -IBD	Hospital -SOT	Hospital Immune -Compromised	Hospital - CKD	Long Term Care	Long Term Acute Care	Community	Total
No. of Susceptible Individuals	19,572,104 (6.13 %)	25,011 (0.01 %)	197,927 (0.06 %)	90,471 (0.03 %)	349,042 (0.11 %)	355,780 (0.11 %)	3,343,595 (1.05 %)	338,094 (0.11 %)	295,154,163 (92.40 %)	319,426,189 (100 %)
No. of Primary CDI cases	131,114 (29.85 %)	1,659 (0.38 %)	6,897 (1.57 %)	2,750 (0.63 %)	14,282 (3.25 %)	13,526 (3.08 %)	112,152 (25.53 %)	6,209 (1.41 %)	150,648 (34.30 %)	439,237 (100 %)
No. of Recurrent CDI Cases	65,664 (39.36 %)	644 (0.39 %)	3,128 (1.88 %)	1,190(0.71 %)	6,843 (4.10 %)	6,730 (4.03 %)	61,247 (36.71 %)	3,064 (1.84 %)	18,311 (10.98 %)	166,821 (100 %)
1st recurrence	36,361 (37.56 %)	392 (0.40 %)	1,791 (1.85 %)	696(0.72 %)	3,847 (3.97 %)	3,736 (3.86 %)	32,970 (34.06 %)	1,705 (1.76 %)	15,309 (15.81 %)	96,807 (100 %)
2nd recurrence	15,756 (41.17 %)	147 (0.38 %)	737 (1.93 %)	278(0.73 %)	1,630 (4.26 %)	1,613 (4.21 %)	14,904 (38.94 %)	734 (1.92 %)	2,474 (6.46 %)	38,273 (100 %)
3rd recurrence	7,096 (42.49 %)	58 (0.35 %)	320 (1.92 %)	117 (0.70 %)	722 (4.32 %)	724 (4.34 %)	6,910 (41.38 %)	329 (1.97 %)	423 (2.53 %)	16,699 (100 %)
4+ recurrences	6,452 (42.88 %)	46 (0.31 %)	280 (1.86 %)	99 (0.66 %)	645 (4.29 %)	657 (4.37 %)	6,464 (42.96 %)	297 (1.97 %)	105 (0.70 %)	15,045 (100 %)
Severe CDI Cases (primary + recurrent)	19,366 (32.59 %)	119 (0.20 %)	790 (1.33 %)	231 (0.39 %)	1,805 (3.04 %)	1,681 (2.83 %)	23,688(39.86 %)	875(1.47 %)	10,876 (18.30 %)	59,431 (100 %)
Deaths Attributable to CDI (primary + recurrent)	14,525 (32.59 %)	89 (0.20 %)	592 (1.33 %)	173 (0.39 %)	1,353 (3.04 %)	1,261 (2.83 %)	17,766 (39.86 %)	656(1.47 %)	8,157 (18.30 %)	44,572 (100 %)

High risk susceptible individuals (i.e., HSCT, IBD, SOT, CKD, other immunocompromised patients) were 5 % of the total hospital population but accounted for 23 % of all CDI patients within hospital. Amongst the 355,780 patients with CKD, over 13,000 patients developed CDI, and burden was similar in patients who were immune compromised. Between 1,600 and 6,900 patients from each of the remaining risk groups (HSCT, IBD, and SOT) developed CDI. However, the majority of patients developing CDI in hospital (131,114; 77.0 %) were not associated with any of the five major risk groups.

Combining primary and all recurrent episodes, 13.5 % of patients had severe CDI, and over 44,500 deaths attributable to CDI were estimated to occur in 2014, corresponding to a 10 % attributable CDI case-fatality.

Approximately one patient in four who was associated with a healthcare setting having a primary case of CDI had at least one further episode, but only 1 in 10 from the community experienced a recurrence. Slightly fewer than one-half of patients who experienced a first recurrent episode had a second recurrence, and about half of those had a third recurrence. Recurrent cases by age are given in the Additional file 1: Appendix C.

Combining all care settings, the greatest disease burden occurs in patients aged 65 years and over, representing 68.6 % of all CDI episodes, while 31.4 % of CDI episodes occurred in those <64 years (Table 2). The majority of CDI cases occurring in patients up to age 44 were in the community. For patients over age 65 years, the majority of CDI cases were found in the hospital or LTC setting (Table 2).

**Table 2:** Model-based estimates of CDI burden in the United States for 2014 by setting and age. Figures in parentheses are proportion of row totals

	1-4 Years	5-9 Years	10-17 Years	18-44 Years	45-64 Years	<64 Years	65-84 Years	≥ 85 Years	Total
Hospital	1,488 (0.58 %)	818 (0.32 %)	1,638 (0.64 %)	18,753 (7.37 %)	56,936 (22.38 %)	79,632 (31.30 %)	122,836 (48.28 %)	51,959 (20.42 %)	254,428 (100 %)
LTC	18 (0.01 %)	17 (0.01 %)	19 (0.01 %)	1,375 (0.79 %)	6,631(3.82 %)	8,059 (4.65 %)	81,857 (47.21 %)	83,482 (48.14 %)	173,399 (100 %)
LTAC	57 (0.61 %)	31 (0.33 %)	62 (0.67 %)	711 (7.67 %)	2,069 (22.31 %)	2,929 (31.58 %)	4,438 (47.85 %)	1,906 (20.55 %)	9,274 (100 %)
Community	2,900 (1.72 %)	3,631 (2.15 %)	6,000 (3.55 %)	34,126 (20.20 %)	52,982 (31.36 %)	99,639 (58.97 %)	59,845 (35.42 %)	9,475 (5.61 %)	168,959 (100 %)
Total	4,463 (0.74 %)	4,497 (0.74 %)	7,719 (1.27 %)	54,965 (9.07 %)	118,618 (19.57 %)	190,259 (31.39 %)	268,976 (44.38 %)	146,822 (24.23 %)	606,060 (100 %)

### Economic burden

Estimated economic costs of CDI in patients in the hospital, LTC, or LTAC were $4.7 billion dollars (86.7 %) while the estimated cost of CDI in the community was $725 million dollars (13.3 %), totaling $5.4 billion (Table 3). Amongst all healthcare resource components, overnight stays in the hospital contributed 78 % of the total direct and indirect costs of CDI cases in healthcare facilities and 52 % of costs for CDI cases originating in the community (Table 3). Costs for pharmacotherapy, healthcare professionals, surgery, and procedures were each under 8 % as a share of total costs in healthcare facilities and under 15 % in the community. Productivity losses associated with inability to work totaled $208 million (based on cases from all settings and initial and recurrent CDI episodes combined). The share of economic burden attributed to either initial or recurrent CDI differed between healthcare facilities and the community. In healthcare facilities, 67 % ($3.2 billion) of costs was due to initial episodes and 33 % ($1.5 billion) was due to recurrent episodes. For initial and recurrent costs, this share was 89 % ($647 million) and 11 % ($77 million), respectively, in the community (see Table 3).

**Table 3:** Economic burden of CDI in non-community (hospital, LTC, LTAC) and community settings in $USD. Figures in parentheses are percentage of total costs

Non-community (Hospital, LTC, LTAC)			
	Cost of Primary CDI Cases ($USD)	Cost of Recurrent CDI ($USD)	Total Cost, Primary and Recurrent ($USD)
Direct Costs Due to CDI	3,077,422,555(65.14 %)	1,515,321,696(32.07 %)	4,592,744,251(97.21 %)
Hospitalization Costs of Initial Cases	2,448,638,121(51.83 %)	1,228,876,113(26.01 %)	3,677,514,233(77.84 %)
Pharmacotherapy Costs of CDI Cases	182,348,058(3.86 %)	111,155,318(2.35 %)	293,503,376(6.21 %)
Healthcare Professional Costs of Initial Cases	271,079,343(5.74 %)	112,255,374(2.38 %)	383,334,717(8.11 %)
Surgical and Medical Procedure Costs	175,357,033(3.71 %)	63,034,891(1.33 %)	238,391,925(5.05 %)
Indirect Costs Due to CDI			
Productivity Costs	92,848,421(1.97 %)	39,010,416(0.83 %)	131,858,837(2.79 %)
Cumulative number of hospital days	1,475,896(0.03 %)	801,821(0.02 %)	2,277,718(0.05 %)
Number of Lost Productivity Days	800,309(0.02 %)	348,256(0.01 %)	1,148,565(0.02 %)
Total Non-Community Cost	3,170,270,976(67.10 %)	1,554,332,112(32.90 %)	4,724,603,089(100.00 %)
Community			
	Cost or Days of Primary CDI($USD or days)	Cost or Days of Recurrent CDI($USD or days)	Total, Primary and Recurrent($USD or days)
Direct Costs Due to CDI	577,985,724(79.78 %)	69,767,755(9.63 %)	647,753,479(89.41 %)
Hospitalization Costs of Initial Cases	337,526,181(46.59 %)	43,205,334(5.96 %)	380,731,515(52.55 %)
Pharmacotherapy Costs of CDI Cases	92,088,119(12.71 %)	13,914,636(1.92 %)	106,002,754(14.63 %)
Healthcare Professional Costs of Initial cases	58,520,790(8.08 %)	5,214,073(0.72 %)	63,734,863(8.80 %)
Surgical and Medical Procedure Costs	89,850,635(12.40 %)	7,433,712(1.03 %)	97,284,347(13.43 %)
Indirect Costs Due to CDI			
Productivity Costs	69,351,125(9.57 %)	7,393,919(1.02 %)	76,745,044(10.59 %)
Cumulative Number of Hospital Days	197,951(0.03 %)	26,213(0.00 %)	224,163(0.03 %)
Number of Lost Productivity Days	602,020(0.08 %)	68,108(0.01 %)	670,128(0.09 %)
Total Community Cost	647,336,849(89.35 %)	77,161,674(10.65 %)	724,498,524(100.00 %)
All settings			
	Cost or Days of Primary CDI($USD or days)	Cost or Days of Recurrent CDI($USD or days)	Total, Primary and Recurrent($USD or days)
Direct Costs Due to CDI	3,655,408,279(67.08 %)	1,585,089,451(29.09 %)	5,240,497,731(96.17 %)
Hospitalization Costs of Initial Cases	2,786,164,301(51.13 %)	1,272,081,447(23.34 %)	4,058,245,748(74.48 %)
Pharmacotherapy Costs of CDI Cases	274,436,177(5.04 %)	125,069,954(2.30 %)	399,506,131(7.33 %)
Healthcare Professional Costs of Initial cases	329,600,133(6.05 %)	117,469,447(2.16 %)	447,069,580(8.20 %)
Surgical and Medical Procedure Costs	265,207,668(4.87 %)	70,468,604(1.29 %)	335,676,272(6.16 %)
Indirect Costs Due to CDI			
Productivity Costs	162,199,547(2.98 %)	46,404,335(0.85 %)	208,603,882(3.83 %)
Cumulative Number of Hospital Days	1,673,847(0.03 %)	828,034(0.02 %)	2,501,881(0.05 %)
Number of Lost Productivity Days	1,402,329(0.03 %)	416,364(0.01 %)	1,818,693(0.03 %)
Total Cost	3,817,607,826(70.06 %)	1,631,493,786(29.94 %)	5,449,101,612(100.00 %)

### Sensitivity analysis

Additional file 1: Appendix C shows combined CDI burden estimates in all settings, age-, and risk-groups for year 2014 under alternative parameter assumptions in one-way sensitivity analyses. Cases of initial CDI were most sensitive to crude incidence of CDI (range 351,389 – 527,084). Recurrent CDI was most sensitive to recurrence rates (range 102,160–261,703), but probability of severe CDI and prevalence of NAP1/BI/027 strain were also influential on total number of recurrent cases. All-cause and attributable deaths were most sensitive to crude incidence of CDI and prevalence of NAP1/BI/027 strain. By varying cost of hospitalization, annual costs of CDI ranged between $3.97 and $6.91 billion dollars in 2014.

### Limitations

As with all models, there were a number of limitations arising from data availability. As our model drew its parameter values from published literature, it was critical to determine that the published data were based on suitable definitions and representative patients that matched the model definitions. This can be problematic when the model requires recurrence, mortality, and healthcare resource use to be conditioned on strain and severity of the episode, a level of detail which is not generally available. This required a very careful selection of data or combinations of data to run the model. Where values from non-US studies were used, it was assumed that the published estimate from Canada and Europe were based on suitably comparable populations. However, a particular strength of the model is its use of HCUP SID data, which permitted estimation of CDI incidence rates by age and high risk groups for HSCT, IBD, SOT, CKD and immune-compromised patients. The estimates of economic burden depended on assumptions surrounding hospitalization cost and length of stay, which due to their uncertainty, led to wide predicted ranges.

Structural and analytic choices were made as in all modeling projects. In particular, our model used a dichotomization of *C*. *difficile* strain and a population stratification that did not include sex and race, which others have found to be important predictors of incidence in the United-States [12]. Another important analytic choice was the calculation of productivity loss that depended on labour participation, which is generally low in patients over 65 years of age who are most affected by CDI. This choice has the effect of reducing the estimated economic burden of CDI compared with estimating the social value of non-workers and retirees as equal to the employed population. Productivity losses for caregivers of children and elderly, which may be important components of economic burden, were also not included due to lack of data.

## Discussion

We developed a decision-analytic model in order to better understand the current and projected CDI burden in the US. Our model allowed calculation of the number of initial and recurrent CDI cases, and mortality rates by age and risk groups and by setting of care. Previously published studies have lacked comparability and this level of granularity, thereby limiting clear interpretation of true burden [10, 12, 15–24, 41]. Crude national estimates of incidence or mortality have not considered the strain of *C. difficile* or provided burden in specific patient populations with serious comorbid conditions.

Our results agree with available estimates from US CDI national surveillance [12]. Our model estimates that in 2014, the absolute annual number of incident CDI cases, when combining all healthcare settings and community-based cases, was over 439,000 cases vs. the Centers for Disease Control and Infection (CDC) figure of 453,000 cases in 2011 [12]. Our model estimates that 25.5 % of these cases originate within an LTC setting, compared to 26 % estimated by CDC. Furthermore, our model estimated that 34.2 % of CDI cases occurred in the community which compares with their figure of 32 % of patients who were defined as community onset and who did not have a hospital stay in the previous 12 weeks. Estimates of first recurrences were reasonably similar: our model estimated 96,807 in 2014 compared to the CDC figure of 83,000 in 2011. However, estimates of deaths differed. Our model estimates that 44,572 deaths occurred in 2014 which is close to the upper limit of CDC estimates of 29,300 (95 % CI, 16,500 – 42,100). One reason explaining this difference is that our model estimated up to 6 recurrences and hence more CDI-attributable deaths were possible in our model, while CDC figures estimated first recurrences only. It is interesting to note that CDI-attributable death estimates from both our model-based estimates and those of CDC exceed figures reported by the National Vital Statistics Reports (NVSS) of 7,994 in 2011. One reason that may explain the discrepancy with NVSS may be that the recorded cause of death in CDI patients with multiple comorbidities were attributed to other causes [42].

Previous estimates of the annual economic cost for CDI in the US ranged from $436 million to $4.8 billion (2008 US dollars) [34–36]. However, it has been suggested that these are low estimates because they do not take into account factors such as outpatient costs, indirect costs, and decreases in productivity [36]. Our estimates, which factored these items, were accordingly higher, indicating a total cost of $5.4 billion annually. In our study, the main driver of costs was hospitalization, accounting for between half to three-quarters of total costs, depending on care setting. Up to one-third of total costs were associated with recurrent CDI for patients whose initial episode was associated with a healthcare facility, but this was much lower when the initial episode was community acquired. Indirect costs were found to be small relative to total economic burden, due to low labor participation in the majority of CDI patients who are over 65 years.

## Conclusions

The results of the model described for this study are useful for better identification of the settings and risk- and age- groups in which CDI burden lies. Moreover, these data permit a greater understanding of the burden of recurrent episodes which has not been reported previously. Given the clinical challenges associated with the of treatment of recurrent CDI and the improved recognition of CDI in high risk groups, these data provide insights into the potential value of emerging new treatment options that could benefit patients with CDI.

## Abbreviations

CDC, Centers for Disease Control and Infection; CDI, *C. difficile* infection; CKD, chronic kidney disease; GW, general ward; HCUP, Healthcare Cost and Utilization Project; HSCT, hematopoietic stem cell transplantation; IBD, inflammatory bowel disease; ICD, International Statistical Classification of Diseases; LTAC, long-term acute care; LTC, long-term care facilities; NAP1, North American Pulsed Field type 1; SID, State Inpatient Databases; SOT, solid organ transplant; US, United States

## Additional file

Additional file 1: Appendix A: Population and Setting. Appendix B: Demographic, epidemiologic and economic model parameters. Appendix C: Supplementary Methods and Results. (DOCX 132 kb)
