# Supplementary material for: Epidemiological and economic burden of Clostridium difficile in the United States: estimates from a modeling approach
Source: BMC Infect Dis. 2016 Jun 18;16:303. doi: 10.1186/s12879-016-1610-3 (PMC4912810; doi:10.1186/s12879-016-1610-3)
Supplement: Additional file 1: — Appendix A: Population and Setting. Appendix B: Demographic, epidemiologic and economic model parameters. Appendix C: Supplementary Methods and Results. (DOCX 132 kb) [file 12879_2016_1610_MOESM1_ESM.docx]

**Appendices to Manuscript “Epidemiological and economic burden of Clostridium difficile in the United-States: estimates from a modelling approach”**

**Appendix A: Population and Setting**

The number of susceptible individuals in each subgroup was derived from multiple sources: US Census Bureau, Centers for Medicare and Medicaid Services (CMS) and HCUPNet.[1-5] Numbers of patients in hospital were derived from HCUPNet which defines hospitals as short-term, non-Federal, general and other hospitals, excluding hospital units of other institutions (e.g., prisons). This includes obstretrics and gynecology, ear, nose, throat, orthopedic, cancer, pediatric, public, and academic medical hospitals. They exclude hospitals whose main focus is long-term care, psychiatric, and alcoholism and chemical dependency treatment. LTC patients consist of those in CMS certified nursing homes in the United States. The number of patients in LTACs followed the CMS’ definition as care in hospitals with a mean length of stay equal to or greater than 25 days.[6] Typically LTACs provide care for patients who do not require all of the services of a short stay hospital but still have significant ongoing care needs. Susceptibles from the community are the non-hospitalized population of the United-States. More specifically they are those who have not had any overnight stay in a healthcare facility in the previous 12 weeks where exposure to *Clostridium difficile* could have occurred.[7,8]

The hospital population was further divided into six groups who are at high risk for CDI: patients with hematopoietic stem cell transplantation (HSCT), inflammatory bowel disease (IBD), solid organ transplant (SOT), severe chronic kidney disease (CKD), immune-compromised patients, and a reference group admitted to hospital not belonging to the previous 5 risk groups. The numbers of such patients were derived from ICD-9-CM codes from HCUP State Inpatient Datasets (SID) databases[9] which provide the absolute number of unique individuals by the defined age bands and risk groups for year 2010 in nine US states, permitting determination of the distribution of these patients which was extrapolated to the total US hospital population. Although medication associated risk factors could define more patient groups at high risk, such as those taking broad-spectrum antibiotics or proton pump inhibitors, we defined our age- and risk-groups according to stratifications that were possible from HCUP databases, a key source of data for the model.[5]

HSCT patient were those admitted to hospital with allogeneic or autologous hematopoietic stem cell transplants, which includes bone marrow, peripheral blood stem cells and cord blood transplants. HCUP SID databases provided absolute number of unique HSCT individuals by age and risk group for year 2010. HSCT patients correspond to ICD9 codes of V42.81, V42.82 and procedure codes 41.00-41.09. It was assumed that patients aged 85+ do not receive stem cell transplantation. Patients with IBD are those with Crohn’s disease and ulcerative colitis, corresponding to ICD-9 codes 555.0-555.9, 556.0-556.9. SOT patients corresponded to ICD-9 diagnosis codes V42.0 – V42.7, V42.83 – V42.89 and ICD-9 procedure codes 33.50-33.52, 37.51, 50.59, 55.69, 33.6, 52.80, 46.97 for solid organ transplant of kidney, liver, pancreas, kidney/pancreas, heart, lung, heart/lung, intestine. ICD-9 codes for severe chronic kidney disease were 403 for hypertensive chronic kidney disease, subcodes .01, .11, .91; 404 for hypertensive heart and chronic kidney disease subcodes .02, .03, .12, .13, .92, .93; and 585.5 and 585.6 for chronic kidney disease. ICD-9 codes for immune compromised patients were 279.01-279.49, 279.8, 279.9 for immune disorders; 288.00-288.2 for white blood cell disorders; 042 for HIV; 262 for malnutrition; 204.00-208.79 for leukaemia/lymphoma. Hospital-based patients not belonging to any of HSCT, IBD, SOT, CKD or immune compromised groups belong to the hospital-reference group.

**Appendix B: Demographic, epidemiologic and economic model parameters**

Table B1. Susceptible pools of patients included in epidemiological model and data sources

|  | Model Base-Case Parameter Values | Source |
| --- | --- | --- |
| Total community population, 2014 | 295,154,163 | US Census Bureau. [1-4] |
| Community age-wise distribution | \| Age \| <1 \| 1-4 \| 5-9 \| 10-17 \| 18-44 \| 45-64 \| 65-84s \| 85+ \| \| --- \| --- \| --- \| --- \| --- \| --- \| --- \| --- \| --- \| \| % \| 1.28 \| 5.25 \| 6.57 \| 10.86 \| 36.52 \| 26.44 \| 11.29 \| 1.79 \| | US Census Bureau . [10] |
| Hospital population, total 2014 | 20,590,383 | HCUPNet [5] and HCUP State Inpatient Databases [9] |
| Hospital age-wise distribution of HSCT patients | \| Age \| <1 \| 1-4 \| 5-9 \| 10-17 \| 18-44 \| 45-64 \| 65-84 \| 85+ \| \| --- \| --- \| --- \| --- \| --- \| --- \| --- \| --- \| --- \| \| % (*) \| 0.0004 \| 0.0030 \| 0.0032 \| 0.0045 \| 0.0268 \| 0.0587 \| 0.0249 \| 0.0000 \| | HCUP State Inpatient Databases [9] |
| Hospital age-wise distribution of IBD patients | \| Age \| <1 \| 1-4 \| 5-9 \| 10-17 \| 18-44 \| 45-64 \| 65-84 \| 85+ \| \| --- \| --- \| --- \| --- \| --- \| --- \| --- \| --- \| --- \| \| % (*) \| 0.0007 \| 0.0023 \| 0.0041 \| 0.0236 \| 0.2853 \| 0.3233 \| 0.2751 \| 0.0469 \| | HCUP State Inpatient Databases [9] |
| Hospital age-wise distribution of SOT patients | \| Age \| <1 \| 1-4 \| 5-9 \| 10-17 \| 18-44 \| 45-64 \| 65-84 \| 85+ \| \| --- \| --- \| --- \| --- \| --- \| --- \| --- \| --- \| --- \| \| % (*) \| 0.0015 \| 0.0056 \| 0.0047 \| 0.0102 \| 0.0868 \| 0.2165 \| 0.1127 \| 0.0014 \| | HCUP State Inpatient Databases [9] |
| Hospital age-wise distribution of CKD patients | \| Age \| <1 \| 1-4 \| 5-9 \| 10-17 \| 18-44 \| 45-64 \| 65-84 \| 85+ \| \| --- \| --- \| --- \| --- \| --- \| --- \| --- \| --- \| --- \| \| % (*) \| 0.0005 \| 0.0008 \| 0.0008 \| 0.0037 \| 0.1732 \| 0.6155 \| 0.7717 \| 0.1617 \| | HCUP State Inpatient Databases [9] |
| Hospital age-wise distribution of immune compromised patients | \| Age \| <1 \| 1-4 \| 5-9 \| 10-17 \| 18-44 \| 45-64 \| 65-84 \| 85+ \| \| --- \| --- \| --- \| --- \| --- \| --- \| --- \| --- \| --- \| \| % (*) \| 0.0105 \| 0.0221 \| 0.0187 \| 0.0283 \| 0.2515 \| 0.5582 \| 0.6203 \| 0.1855 \| | HCUP State Inpatient Databases [9] |
| Hospital age-wise distribution of reference group. | \| Age \| <1 \| 1-4 \| 5-9 \| 10-17 \| 18-44 \| 45-64 \| 65-84 \| 85+ \| \| --- \| --- \| --- \| --- \| --- \| --- \| --- \| --- \| --- \| \| % (*) \| 5.4644 \| 1.0992 \| 0.7972 \| 2.1622 \| 29.0523 \| 23.6380 \| 24.9203 \| 7.9211 \| | HCUP State Inpatient Databases [9] |
| LTC patients, 2014 | 3,343,592 | CMS Nursing Home Compendium [11] |
| LTC age-wise distribution | \| Age \| <1 \| 1-4 \| 5-9 \| 10-17 \| 18-44 \| 45-64 \| 65-84 \| 85+ \| \| --- \| --- \| --- \| --- \| --- \| --- \| --- \| --- \| --- \| \| % \| 0.0091 \| 0.0364 \| 0.0455 \| 0.0727 \| 5.9776 \| 8.0588 \| 48.9 \| 36.9 \| | CMS Nursing Home Compendium [11] |
| Total LTAC patients, 2014 | 338,094 | Kahn et al, 2010 [6,9] |
| LTAC age-wise distribution | \| Age \| <1 \| 1-4 \| 5-9 \| 10-17 \| 18-44 \| 45-64 \| 65-84 \| 85+ \| \| --- \| --- \| --- \| --- \| --- \| --- \| --- \| --- \| --- \| \| % \| 5.75 \| 1.16 \| 0.84 \| 2.27 \| 30.56 \| 24.87 \| 26.22 \| 8.33 \| | Assumed to be same as hospital reference. |

(*) Percentages are over total hospital population

Table B2. Epidemiologic parameters and data sources

|  | Model Base-Case Parameter Values | Source |
| --- | --- | --- |
| Hospital, crude CDI incidence of initial episode (per 1000 admissions), 2014 | 8.46 | Lucado et al., 2009 [12];  HCUP SID analyses ([9]) Pawar et al., 2012 ([13]); Chitnis et al, 2013 ([14]) |
| Hospital age-wise CDI incidence of HSCT patients in 2010 (*) | \| Age \| <1 \| 1-4 \| 5-9 \| 10-17 \| 18-44 \| 45-64 \| 65-84 \| 85+ \| \| --- \| --- \| --- \| --- \| --- \| --- \| --- \| --- \| --- \| \| (***) \| 0.0 \| 16.8 \| 7.4 \| 11.6 \| 8.3 \| 7.6 \| 6.3 \| 10.9 \| | HCUP State Inpatient Databases [9] |
| Hospital age-wise CDI incidence of IBD patients in 2010(*) | \| Age \| <1 \| 1-4 \| 5-9 \| 10-17 \| 18-44 \| 45-64 \| 65-84 \| 85+ \| \| --- \| --- \| --- \| --- \| --- \| --- \| --- \| --- \| --- \| \| (***) \| 0.0 \| 2.7 \| 3.3 \| 5.0 \| 2.9 \| 3.1 \| 5.6 \| 9.7 \| | HCUP State Inpatient Databases [9] |
| Hospital age-wise CDI incidence of SOT patients in 2010(*) | \| Age \| <1 \| 1-4 \| 5-9 \| 10-17 \| 18-44 \| 45-64 \| 65-84 \| 85+ \| \| --- \| --- \| --- \| --- \| --- \| --- \| --- \| --- \| --- \| \| (***) \| 0.0 \| 7.2 \| 5.6 \| 1.7 \| 2.9 \| 3.4 \| 4.5 \| 3.5 \| | HCUP State Inpatient Databases [9] |
| Hospital age-wise CDI incidence of CKD patients in 2010 (*) | \| Age \| <1 \| 1-4 \| 5-9 \| 10-17 \| 18-44 \| 45-64 \| 65-84 \| 85+ \| \| --- \| --- \| --- \| --- \| --- \| --- \| --- \| --- \| --- \| \| (***) \| 0.0 \| 4.5 \| 0.0 \| 0.7 \| 2.8 \| 3.9 \| 5.2 \| 5.4 \| | HCUP State Inpatient Databases [9] |
| Hospital age-wise CDI incidence of immune compromised patients in 2010 (*) | \| Age \| <1 \| 1-4 \| 5-9 \| 10-17 \| 18-44 \| 45-64 \| 65-84 \| 85+ \| \| --- \| --- \| --- \| --- \| --- \| --- \| --- \| --- \| --- \| \| (***) \| 0.0 \| 4.8 \| 4.5 \| 5.0 \| 3.3 \| 4.0 \| 5.8 \| 6.4 \| | HCUP State Inpatient Databases [9] |
| Hospital age-wise CDI incidence of patients not having above comorbidities in 2010 (*) | \| Age \| <1 \| 1-4 \| 5-9 \| 10-17 \| 18-44 \| 45-64 \| 65-84 \| 85+ \| \| --- \| --- \| --- \| --- \| --- \| --- \| --- \| --- \| --- \| \| (***) \| 0.0 \| 0.4 \| 0.3 \| 0.2 \| 0.2 \| 0.7 \| 1.4 \| 2.1 \| | HCUP State Inpatient Databases [9] |
| LTC, crude incidence (per 1000 residents), 2014 | 18.6 | Campbell, et al., 2009 [15]; Laffan, et al., 2006 [16] |
| LTC, CDI incidence by age- of initial episode | \| Age \| <1 \| 1-4 \| 5-9 \| 10-17 \| 18-44 \| 45-64 \| 65-84 \| 85+ \| \| --- \| --- \| --- \| --- \| --- \| --- \| --- \| --- \| --- \| \| (***) \| 0.0 \| 0.6 \| 0.5 \| 0.3 \| 0.3 \| 0.9 \| 1.7 \| 2.4 \| | Assumed age-wise incidence estimated with HCUP SID analyses due to lack of LTC-specific data. |
| LTAC, crude incidence (per 1000 residents), 2014 | 18.6 | Due to a lack of data on CDI incidence in LTAC patients, we assumed the same incidence rates as for LTC. |
| LTAC, CDI incidence by age- of initial episode | \| Age \| <1 \| 1-4 \| 5-9 \| 10-17 \| 18-44 \| 45-64 \| 65-84 \| 85+ \| \| --- \| --- \| --- \| --- \| --- \| --- \| --- \| --- \| --- \| \| Inc.(***) \| 0.0 \| 11.3 \| 8.5 \| 6.3 \| 5.4 \| 17.4 \| 32.0 \| 43.8 \| | Due to a lack of data on CDI incidence in LTAC patients, we assumed the same incidence rates as for LTC. |
| Community, crude incidence (per 1000 population),2014 | 0.35 | Lessa, ID Week, 2012 [17]; Kutty et al. 2010 [18] |
| Community, CDI incidence by age- of initial episode in 2010 | \| Age \| <1 \| 1-4 \| 5-9 \| 10-17 \| 18-44 \| 45-64 \| 65-84 \| 85+ \| \| --- \| --- \| --- \| --- \| --- \| --- \| --- \| --- \| --- \| \| Inc.(**) \| 0.00 \| 0.17 \| 0.17 \| 0.17 \| 0.29 \| 0.61 \| 1.56 \| 1.56 \| | Lessa, ID Week, 2012 [17] |
| Probability infection with NAP1/BI/027 | 0.28 | Lessa, ID Week, 2012 [17] |
| Probability of severe CDI | Infecting strain NAP1/BI/027   \| Age \| <1 \| 1-4 \| 5-9 \| 10-17 \| 18-44 \| 45-64 \| 65-84 \| 85+ \| \| --- \| --- \| --- \| --- \| --- \| --- \| --- \| --- \| --- \| \| Prob. \| 0.064 \| 0.064 \| 0.064 \| 0.064 \| 0.064 \| 0.075 \| 0.125 \| 0.313 \|   Infecting strain non-NAP1/BI/027   \| Age \| <1 \| 1-4 \| 5-9 \| 10-17 \| 18-44 \| 45-64 \| 65-84 \| 85+ \| \| --- \| --- \| --- \| --- \| --- \| --- \| --- \| --- \| --- \| \| Prob. \| 0.030 \| 0.030 \| 0.030 \| 0.030 \| 0.030 \| 0.035 \| 0.059 \| 0.148 \| | Miller et al. 2010 [19];  Khanna et al., 2012 [20]  Gravel et al., 2009 [21];  Bauer et al., 2011 [22];  Loo et al., 2011 [23];  McGowan et al., 2011 [24] |
| Mortality & treatment outcome | Mortality, non-severe CDI = 0.13  Survival with treatment with 1^st^ course antibiotic, non-severe CDI = 0.7  Survival with treatment with additional courses antibiotics, non-severe CDI = 0.17  Mortality, severe CDI = 0.75  Survival with treatment with 1^st^ course antibiotics, severe CDI = 0.08  Survival with treatment with additional course antibiotics, severe CDI = 0.17 | Khanna et al., 2012 [20]  Gravel et al., 2009 [21]  Bauer et al., 2011 [22]  Loo et al., 2011 [23]  McGowan, et al., 2011 [24]  Wiegand, et al , 2012 [25] |
| Recurrence | All settings except community:  1st, non-NAP1, age 0-44 0.2  2nd +, non-NAP1, age 0-44 0.3  1st, NAP1, age 0-44 0.3  2nd +, NAP1, age 0-44 0.45  Community:  1st, non-NAP1, age 0-44 0.081  2nd +, non-NAP1, age 0-44 0.121  1st, NAP1, age 0-44 0.121  2nd +, NAP1, age 0-44 0.181  Relative Risk of Recurrence for older age groups  age 45-64 1.3  age 65-84 1.6  age 85+ 1.75 | Johnson et al., 2009; [26]; Lee BY et al, 2010; [27]; Loo, et al., 2011. [23]; Laffan, et al., 2006 (6); Khanna, et al., 2012 [20]; Kelly CP. 2012. [28]; Wiegand, et al., 2012 [25]; Louie, et al., , 2013 [29]; Eyre et al. 2012 [30]; Lessa, ID Week, 2012 [17] |

(*) Figures given were estimated from 2010 data from HCUP State Inpatient Databases and were assumed to apply to year 2014.

(***) Figures given are multiplicative factors for incidence for a specific risk and age group that applies to the crude incidence. For example, CDI incidence in HSCT patients aged 85+ in 2014 is 10.9 * 8.46 = 92.2 per 1000 admissions. CDI incidence in LTC patients aged 18-44 is 0.3 * 18.6 = 5.58 per 1000 residents.

In the base-case, all non-severe cases of CDI incurred 3 bed-days of hospitalization in the general ward (GW)[31-34] costing $1,744 per day for patients aged 0-64 yrs or $1,590 for those over 65 yrs.[5,35] It was assumed that a severe case of CDI required 12 bed-days in the general ward plus 11 bed-days in an intensive care unit (ICU).[36] While all non-severe hospital, LTC or LTAC patients incurred GW bed-day costs, only 17.3% of CDI cases from community incurred costs for bed-days.[14] Unit bed-day costs reflect the actual expenses incurred in the production of hospital services, such as nurses wages, supplies, and utility costs but do not include physician fees or pharmacotherapy costs. Use and quantity of pharmacotherapy such as oral metronidazole, oral vancomycin, tapered vancomycin, fidaxomicin, or vancomycin with intravenous metronidazole was estimated based on current treatment guidelines.[7] The percentage of patients receiving each therapeutic regimen varied according to CDI episode and disease severity and was estimated using unpublished data from Phase III efficacy trials currently being conducted of *C.difficile* monoclonal antibodies (Trial Identifier ClinicaTrials.gov: NCT01241552). Contact with healthcare professionals in the hospital, LTC or LTAC setting for the management of nonsevere CDI assumed one contact per day valued at $133.50 per contact in the GW and contacts with two separate specialists daily in the ICU valued at $177.08 per contact.[37] CDI cases managed in the community assumed one GP contact per episode costing $76.69 per visit.[37] Costs were also estimated for other procedures including colectomy, abdominal CT scans, peripheral intravenous line insertion, endoscopy and toxin tests.

Productivity losses arising from CDI were calculated according to the product of the following 3 quantities: 1) duration of the episode, assumed to be 7.6 days for non-severe and 23 days for severe CDI;[38] 2) the mean earnings by age of the patient (0-17 yrs, 18-64 yrs, 65+ yrs); and 3) labor participation by age, obtained from the Bureau of Labor Statistics.[35]

Table B3 Economic model parameters and data sources

|  | Model Base-Case Parameter Values | Sources |
| --- | --- | --- |
| Cost GW bed-day | $1,744 for 0-64 yrs  $1,590 for 65+ yrs | Hospital costs from HCUPNet [5]. Inflation index on BLS [35] |
| GW utilization | 3 days for non-severe cases  12 days for severe cases | Song et al. 2008 [34]; Kyne et al. 2002 [32], O’Brien et al. 2007 [33]; Dubberke et al. 2009 [31]; Zilberberg et al. 2009 [36]. |
| ICU cost bed-day | $1,744 for 0-64 yrs  $1,590 for 65+ yrs | Assumed same as GW bed-day. As alternative in sensitivity analysis  an ICU bed-day was 3.6 times that of a GW bed-day (McGlone et al. 2012) [39] |
| ICU utilization | 0 days for non-severe cases  11 days for severe cases | Zilberberg et al. 2009 [36] |
| Metronidazole PO cost | $6.00 per course | Course of oral metronidazole assumed to be 500 mg every 8h for 10 to 14 days (we assumed 10 days) at a cost of $0.60 per day. [40] |
| Metronidazole PO utilization | 57% patents for primary, nonsevere  38% patients for primary, severe  31% patients for 1^st^ recurrence, nonsevere  38% patients for 1^st^ recurrence, severe  13% patients for 2^nd^ + recurrence | Merck trial data (data on file) |
| Vancomycin PO cost | $584.10 per course | Course of oral vancomycin assumed 125 mg every 6h for 10-14 days (we assumed 10 days) at a cost of $58.41 per day[40] |
| Vancomycin PO utilization | 38% patents for primary, nonsevere  48% patients for primary, severe  62% patients for 1st recurrence, nonsevere  48% patients for 1st recurrence, severe  23% patients for 2nd + recurrence | Merck trial data (data on file) |
| Vancomycin tapered cost | $1255.60 per course[40] | Course of tapered vancomycin assumed 4 times a day for 14 days, 2 times a day for 7 days, once a day for 7 days, once every  2 days for 8 days, once every 3 days for 15 days. |
| Vancomycin tapered utilization | 0% patents for primary  0% patients for 1st recurrence  33.1% patients for 2nd + recurrence | Merck trial data (data on file) |
| Fidaxomycin cost | $2589.40 per course | Course of oral fidoxamycin assumed 200 mg orally, 2 times per day for 10 days at a cost of $258.94 per day[40] |
| Fidaxomycin utilization | 1% patents for primary, nonsevere  3% patients for primary, severe  4% patients for 1st recurrence, nonsevere  4% patients for 1st recurrence, severe  26.9% patients for 2nd + recurrence | Merck trial data (data on file) |
| Vancomycin PO + Metronidazole IV cost | $4704.00 per course[40] | Course of oral vancomycin plus intravenous metronidazole assumed vancomycin PO 500 mg orally four times a day and metronidazole 500 mg IV every 8 h and vancomycin per rectum (vancomycin 500 mg in 500 ml saline as enema) four times a day for 10 days. |
| Vancomycin PO + Metronidazole IV utilization | 7% patents for primary, nonsevere  15% patients for primary, severe  6% patients for 1st recurrence, nonsevere  15% patients for 1st recurrence, severe  11% patients for 2nd + recurrence | Merck trial data (data on file) |
| HCP in general ward | $133.50 per contact for hospitalization in GW | CPT code 99235 [37] |
| HCP utilization | 3 contacts non-severe cases  12 contacts severe cases | Assume one contact per day of inpatient stay |
| HCP in ICU | $177.08 per contact for hospitalization in ICU | CPT code 99291 [37] |
| HCP utilization in ICU | 0 contacts non-severe cases  22 contacts severe cases | Assume two contacts per day of ICU stay |
| GP office visit cost (nonsevere community cases) | $76.69 | CPT code 99203, nonfacility rate [37] |
| GP office visit utilization (nonsevere community cases) | 82.7% of nonsevere CDI cases in community are handled by GP with 1 GP contact per patient | Chitnis et al, 2013 [14] |
| Colectomy | $25,907 | Cost represents $23760 obtained from HCUPNet and inflated from 2012 plus $1800 physician fee schedule [5] |
| Colectomy utilization | 0% patents for nonsevere  1% patients for severe | Assumed. |
| Abdominal CT Scan cost | $294.97 | McGlone, citing American Medical Association. CPT code/relative value search, 2010 costs inflated to 2013 [39] |
| Abdominal CT scan utilization | 100%, all patients once per episode |  |
| Peripheral Intravenous Line Insertion cost | $103.61 | McGlone, citing American Medical Association. CPT code/relative value search, 2010 costs inflated to 2013[39] |
| Peripheral Intravenous Line Insertion utilization | 100%, all patients one per episodes | Assumed |
| Endoscopy cost | $665.50 | CPT code 44377 [37] |
| Endoscopy utilization | 25% patents for primary episodes  0% patients for recurrent episodes | McFarland et al (1999) [38] |
| Toxin test cost | $17.24 | CPT code 87493 [37] |
| Toxin test utilization | 100%, all patients one per episode |  |
| Productivity Loss (Patient time cost) | $0 for 0-17 yrs, assumed  $114.77 per day for 18-64 yrs applied to 76.5% (labor participation)  $117.25 per day for 65+ applied to 19.0% (labor participation) | BLS data [35] |
| Patient time quantity | 7.6 days per nonsevere episode  23 days per severe episode | McFarland 1999 [38] |

**Appendix C: Supplementary Methods and Results**

**Table C1. Primary and recurrent CDI cases in the United States by age group for 2014 in all settings (Hospital, LTC, LTAC, community)**

|  | 1 -4 Years | 5-9 Years | 10-17 Years | 18-44 Years | 45-64 Years | <64 Years | 65-84 Years | >=85 Years |
| --- | --- | --- | --- | --- | --- | --- | --- | --- |
| Primary CDI cases | 3,888 | 4,014 | 6,860 | 47,685 | 93,858 | 156,306 | 185,772 | 97,159 |
| No. of Recurrent CDI cases | 573 | 481 | 858 | 7,280 | 24,760 | 33,953 | 83,204 | 49,664 |
| Recurrent cases (1st recurrence) | 442 | 389 | 686 | 5,568 | 16,303 | 23,387 | 46,160 | 27,260 |
| Recurrent cases (2nd recurrence) | 95 | 70 | 129 | 1,228 | 5,191 | 6,712 | 19,549 | 12,011 |
| Recurrent cases (3rd recurrence) | 25 | 16 | 30 | 326 | 1,896 | 2,292 | 8,960 | 5,446 |
| Recurrent cases (4+ recurrences) | 12 | 7 | 14 | 159 | 1,370 | 1,562 | 8,535 | 4,947 |
| Total CDI Cases | 4,461 | 4,496 | 7,719 | 54,965 | 118,618 | 190,259 | 268,976 | 146,823 |

Sensitivity analyses

Crude incidence of initial CDI was assumed +/-20% from the base-case in all settings and year, corresponding roughly to the regional variation of CDI in the US.[41] The probability of NAP1/BI/027 strain was varied between 18%[42] to 82% observed during a 2004 outbreak.[23] Severe CDI varied from 8% to 28% (applied equally to both strain types), based on ranges observed in the literature.[20-24] Mortality ranged from 67% to 90.9% for severe CDI and 11.4% or 15.3% for non-severe, also derived from literature.[20-25] Relative to the base-case, recurrence was incremented by +/-5% for the 1^st^ recurrence and +/-10% for subsequent recurrences.[16,20,23,26-28] Uncertainty for economic outcomes was varied only by the cost of a GW or ICU stay by +/-20% from the base-case. Sensitivity analyses also considered how CDI could vary in future years by varying future number of total susceptible patients in all settings based on annual population growth rates from low and high alternative net migration series[1] and future incidence of CDI for which we assumed a 30% decrease from 2015 levels achieved by 2020, in line with national action plans to reduce CDI,[43]or 30% higher in the same time frame.

**Table C2. Results of one-way sensitivity analysis for years 2014 and 2020** **for all settings, age- and risk-groups combined**

|  | Low Range, Year 2014 | | | | High Range, Year 2014 | | | |
| --- | --- | --- | --- | --- | --- | --- | --- | --- |
|  | Primary CDI | Recurrent CDI | All-cause deaths | Attributable deaths | Primary CDI | Recurrent CDI | All-cause deaths | Attributable deaths |
| Number total susceptible patients in all settings (community, LTC, LTAC, hospital) | 438,351 | 166,564 | 115,444 | 44,499 | 439,799 | 167,039 | 115,803 | 44,630 |
| Crude incidence of CDI | 351,389 | 133,457 | 92,523 | 35,659 | 527,084 | 200,185 | 138,785 | 53,488 |
| Future incidence of CDI | 439,236 | 166,821 | 115,654 | 44,573 | 439,236 | 166,821 | 115,654 | 44,573 |
| Prevalence NAP1/BI/027 strain | 439,236 | 156,175 | 110,333 | 39,807 | 439,236 | 224,308 | 144,387 | 70,308 |
| Probability of severe CDI | 439,236 | 119,301 | 166,782 | 113,883 | 439,236 | 178,074 | 123,423 | 52,203 |
| Mortality | 439,236 | 177,339 | 102,004 | 40,852 | 439,236 | 153,412 | 132,518 | 52,198 |
| Recurrence | 439,236 | 102,160 | 102,805 | 39,211 | 439,236 | 261,703 | 134,571 | 52,519 |
| Cost assumptions | $USD 3,971,219,355 | | | | $USD 6,917,360,596 | | | |
|  |  | | | |  | | | |

Reference List

1. **2012 National Population Projections. Projected Population by Single Year of Age, Sex, Race, and Hispanic Origin for the United States: 2012 to 2060. Middle Series** [<http://www.census.gov/population/projections/data/national/2012/downloadablefiles.html>]

2. **Intercensal Estimates of the Resident Population by Sex and Age for the United States: April 1, 2000 to July 1, 2010 (US-EST00INT-01)** [<http://www.census.gov/popest/data/intercensal/national/nat2010.html>]

3. **Annual Estimates of the Population for the United States, Regions, States, and Puerto Rico: April 1, 2010 to July 1, 2012 (NST-EST2012-01)** [<http://www.census.gov/popest/data/historical/2010s/vintage_2012/national.html>]

4. **Annual Estimates of the Resident Population by Single Year of Age and Sex for the United States: April 1, 2010 to July 1, 2013**  [<http://www.census.gov/popest/data/datasets.html>]

5. **HCUPnet** [<http://hcupnet.ahrq.gov/>]

6. Kahn JM, Benson NM, Appleby D, Carson SS, Iwashyna TJ. Long-term acute care hospital utilization after critical illness**.** JAMA. 2010,303**:**2253-9.

7. Cohen SH, Gerding DN, Johnson S, Kelly CP, Loo VG, McDonald LC, Pepin J, Wilcox MH. Clinical practice guidelines for Clostridium difficile infection in adults: 2010 update by the Society for Healthcare Epidemiology of America (SHEA) and the Infectious Diseases Society of America (IDSA)**.** Infect Control Hosp Epidemiol. 2010,31**:**431-55.

8. **Risk adjustment for healthcare facility-onset C.difficile and MRSA bacteremia laboratory-identified event reporting in NHSN**

9. HCUPnet. Primary analyses conducted with state inpatient databases**.** 2015.

10. **2013 Population Estimates** [<http://factfinder.census.gov/faces/nav/jsf/pages/searchresults.xhtml?refresh=t#none>]

11. **Nursing home data compendium 2010 edition**

12. Lucado J, Gould C, Elixhauser A.: *Clostridium difficile Infections (CDI) in Hospital Stays, 2009.* 2009.

13. Pawar D, Tsay R, Nelson DS, Elumalai MK, Lessa FC, Clifford ML, Dumyati G. Burden of Clostridium difficile infection in long-term care facilities in Monroe County, New York**.** Infect Control Hosp Epidemiol. 2012,33**:**1107-12.

14. Chitnis AS, Holzbauer SM, Belflower RM, Winston LG, Bamberg WM, Lyons C, Farley MM, Dumyati GK, Wilson LE, Beldavs ZG et al. Epidemiology of Community-Associated Clostridium difficile Infection, 2009 Through 2011**.** JAMA Intern Med. 2013,1-9.

15. Campbell RJ, Giljahn L, Machesky K, Cibulskas-White K, Lane LM, Porter K, Paulson JO, Smith FW, McDonald LC. Clostridium difficile Infection in Ohio Hospitals and Nursing Homes During 2006**.** Infect Control Hosp Epidemiol. 2009,30**:**526-33.

16. Laffan AM, Bellantoni ME, Greenough WB, Zenilman JM. Burden of Clostridium difficile-associated diarrhea in a long-term care facility**.** J Am Geriatr Soc. 2006,54**:**1068-73.

17. Lessa FC: **Incidence and insights into C. difficile infection epidemiology [abstract].** *Presented at: ID Week, Oct 18, 2012, San Diego, CA;* 2012,

18. Kutty PK, Woods CW, Sena AC, Benoit SR, Naggie S, Frederick J, Evans S, Engel J, McDonald LC. Risk factors for and estimated incidence of community-associated Clostridium difficile infection, North Carolina, USA**.** Emerg Infect Dis. 2010,16**:**197-204.

19. Miller MA, Kuijper E, Gerding DN, Gorbach SL: **Three Simple ESCMID Severity Criteria Predict Poor Cure Rate and Slower Resolution of Diarrhea in Clostridium Difficile Infection (CDI) [abstract].** *Poster presented at ECCMID, April 10-13, 2010; Vienna Austria* 2010,

20. Khanna S, Pardi DS, Aronson SL, Kammer PP, Orenstein R, St Sauver JL, Harmsen WS, Zinsmeister AR. The epidemiology of community-acquired Clostridium difficile infection: a population-based study**.** Am J Gastroenterol. 2012,107**:**89-95.

21. Gravel D, Miller M, Simor A, Taylor G, Gardam M, McGeer A, Hutchinson J, Moore D, Kelly S, Boyd D et al. Health Care-Associated Clostridium difficile Infection in Adults Admitted to Acute Care Hospitals in Canada: A Canadian Nosocomial Infection Surveillance Program Study**.** Clin Infect Dis. 2009,48**:**568-76.

22. Bauer MP, Notermans DW, van Benthem BH, Brazier JS, Wilcox MH, Rupnik M, Monnet DL, van Dissel JT, Kuijper EJ. Clostridium difficile infection in Europe: a hospital-based survey**.** Lancet. 2011,377**:**63-73.

23. Loo VG, Bourgault AM, Poirier L, Lamothe F, Michaud S, Turgeon N, Toye B, Beaudoin A, Frost EH, Gilca R et al. Host and pathogen factors for Clostridium difficile infection and colonization**.** N Engl J Med. 2011,365**:**1693-703.

24. McGowan AP, Lalayiannis LC, Sarma JB, Marshall B, Martin KE, Welfare MR. Thirty-day mortality of Clostridium difficile infection in a UK National Health Service Foundation Trust between 2002 and 2008**.** J Hosp Infect. 2011,77**:**11-5.

25. Wiegand PN, Nathwani D, Wilcox MH, Stephens J, Shelbaya A, Haider S. Clinical and economic burden of Clostridium difficile infection in Europe: a systematic review of healthcare-facility-acquired infection**.** J Hosp Infect. 2012,81**:**1-14.

26. Johnson S. Recurrent Clostridium difficile infection: A review of risk factors, treatments, and outcomes**.** J Infect. 2009,58**:**403-10.

27. Lee BY, Popovich MJ, Tian Y, Bailey RR, Ufberg PJ, Wiringa AE, Muder RR. The potential value of Clostridium difficile vaccine: an economic computer simulation model**.** Vaccine. 2010,28**:**5245-53.

28. Kelly CP. Can we identify patients at high risk of recurrent Clostridium difficile infection? Clin Microbiol Infect. 2012,18 Suppl 6**:**21-7.

29. Louie TJ, Miller MA, Crook DW, Lentnek A, Bernard L, High KP, Shue YK, Gorbach SL. Effect of age on treatment outcomes in Clostridium difficile infection**.** J Am Geriatr Soc. 2013,61**:**222-30.

30. Eyre DW, Walker AS, Wyllie D, Dingle KE, Griffiths D, Finney J, O'Connor L, Vaughan A, Crook DW, Wilcox MH et al. Predictors of first recurrence of clostridium difficile infection: Implications for initial management**.** Clin Infect Dis. 2012,55**:**S77-S87.

31. Dubberke ER, Wertheimer AI. Review of Current Literature on the Economic Burden of Clostridium difficile Infection**.** Infect Control Hosp Epidemiol. 2009,30**:**57-66.

32. Kyne L, Hamel MB, Polavaram R, Kelly CNP. Health care costs and mortality associated with nosocomial diarrhea due to Clostridium difficile**.** Clin Infect Dis. 2002,34**:**346-53.

33. O'Brien JA, Lahue BJ, Caro JJ, Davidson DM. The emerging infectious challenge of clostridium difficile-associated disease in Massachusetts hospitals: Clinical and economic consequences**.** Infect Control Hosp Epidemiol. 2007,28**:**1219-27.

34. Song XY, Bartlett JG, Speck K, Naegeli A, Carroll K, Perl TM. Rising economic impact of Clostridium difficile - Associated disease in adult hospitalized patient population**.** Infect Control Hosp Epidemiol. 2008,29**:**823-8.

35. **Consumer Price Index inflation calculator** [<http://www.bls.gov/data/inflation_calculator.htm>]

36. Zilberberg MD. Clostridium difficile-related hospitalizations among US adults, 2006**.** Emerg Infect Dis. 2009,15**:**122-4.

37. **Arizona Health Care Cost Containment System, Physician Fee Schedule** [<http://www.azahcccs.gov/commercial/ProviderBilling/rates/Physicianrates/Physicianrates.aspx>]

38. McFarland LV, Surawicz CM, Rubin M, Fekety R, Elmer GW, Greenberg RN. Recurrent Clostridium difficile disease: Epidemiology and clinical characteristics**.** Infect Control Hosp Epidemiol. 1999,20**:**43-50.

39. McGlone SM, Bailey RR, Zimmer SM, Popovich MJ, Tian Y, Ufberg P, Muder RR, Lee BY. The economic burden of Clostridium difficile**.** Clin Microbiol Infect. 2012,18**:**282-9.

40. **Analysource: Suite of Drug Pricing Services** [<https://www.analysource.com/>]

41. Lessa FC, Mu Y, Bamberg WM, Beldavs ZG, Dumyati GK, Dunn JR, Farley MM, Holzbauer SM, Meek JI, Phipps EC et al. Burden of Clostridium difficile infection in the United States**.** N Engl J Med. 2015,372**:**825-34.

42. Davies KA, Longshaw CM, Davis GL, Bouza E, Barbut F, Barna Z, Delmee M, Fitzpatrick F, Ivanova K, Kuijper E et al. Underdiagnosis of Clostridium difficile across Europe: the European, multicentre, prospective, biannual, point-prevalence study of Clostridium difficile infection in hospitalised patients with diarrhoea (EUCLID)**.** Lancet Infect Dis. 2014,14**:**1208-19.

43. **Progress toward eliminating healthcare-associated infections.** [<http://www.health.gov/hai/prevent_hai.asp#CDI>]
